# Analysis of refractive development characteristics in school-age children based on biometric measurements: a cross-sectional study involving 12,025 primary school students from Xingtai City

**DOI:** 10.3389/fpubh.2025.1660018

**Published:** 2025-09-22

**Authors:** Jie Gao, Lizhu Meng, Xi Lv, Ye Yang, Yanyan Liang

**Affiliations:** ^1^Department of Ophthalmology, Shijiazhuang Aier Eye Hospital, Shijiazhuang, China; ^2^Department of Ophthalmology, Xingtai Aier Eye Hospital, Xingtai, China; ^3^Department of Ophthalmology, Hebei General Hospital, Shijiazhuang, China; ^4^Department of Ophthalmology, The Forth Hospital of Hebei Medical University, Shijiazhuang, China; ^5^Department of Ophthalmology, The First Hospital of Hebei Medical University, Shijiazhuang, China

**Keywords:** myopia screening, myopia prevention and control, axial length, keratometry value, vision correction

## Abstract

**Objective:**

To investigate refractive development, myopia prevalence trends, and correction status among 6–12-year-old primary students in Xingtai, Hebei, China, and provide evidence for childhood myopia interventions.

**Methods:**

This cross-sectional study enrolled 12,025 eligible students (6–12 years) from 11 schools (2022–2023). Refractive status was assessed via non-cycloplegic autorefraction (NIDEK AR-1), with ocular biometry [axial length (AL), keratometry (K)]. Diagnostic criteria: spherical equivalent (SE) < −0.50D (myopia), *K* ≤ −0.50D (astigmatism), uncorrected/corrected visual acuity <5.0/<4.9 (subnormal vision/insufficient correction).

**Results:**

Visual impairment prevalence was 65.65% (7,895/12,025), rising significantly from 58.00% (Grade 1) to 75.75% (Grade 6). Myopia prevalence increased from 30.41% to 68.78% (overall 51.69%). SE shifted myopically (−1.46 ± 1.84D overall; Grade 1: −0.66 ± 1.54D vs. Grade 6: −2.20 ± 2.01D). AL increased with grade (23.64 ± 1.07 mm overall; 22.95 ± 0.83 mm to 24.13 ± 1.12 mm), while corneal curvature remained stable (43.39 ± 1.51D; inter-grade variation <0.13D). The AL/corneal radius ratio correlated linearly with SE (3.02 ± 0.17 overall; 2.95 ± 0.10 to 3.08 ± 0.14).

**Conclusion:**

Vision impairment and myopia prevalence show higher prevalence in older age groups in Xingtai primary students. Suboptimal refractive correction rates highlight clinical challenges. AL progression and stable corneal curvature suggest axial elongation drives myopia. The AL/corneal radius ratio’s correlation with SE underscores its potential as a predictor for myopia progression, aiding risk prediction model development.

## Introduction

Childhood myopia has emerged as a critical global public health challenge, marked by an accelerating trend of earlier onset and increasing severity. As reported in the literature (1), the prevalence of myopia among children and adolescents aged 6–19 years exhibits substantial regional disparities. Specifically, the prevalence is approximately 60% in Asia, which is notably higher than the 40% observed in Europe. Projections indicate that by 2050, the global number of individuals with myopia will reach 4.758 billion, including 938 million with high myopia (2). Notably, the rate of increase in myopia prevalence among Chinese children and adolescents surpasses that of other regions. It is estimated that by 2050, the myopia prevalence among Chinese children and adolescents aged 3–19 years will approach 84% (3). An increasing number of researchers have focused on the genetic pathogenesis of myopia in recent years. Scleral remodeling and excessive axial elongating induced retina thinning and even retinal detachment are myopia’s most important pathological manifestations (4, 5). Furthermore, a prospective cohort study conducted in Singapore revealed that early-onset myopia is strongly associated with abnormal axial elongation of the eye, significantly elevating the risk of severe complications such as macular degeneration (6). These findings highlight the urgent need to advance the prevention and control of high myopia to the preschool stage.

The development of myopia is influenced by the complex interplay between genetic predispositions and environmental exposures. Epidemiological studies have consistently shown that the prevalence of myopia among urban children is significantly higher than that among rural children (7, 8). This disparity may be attributed to factors such as prolonged near-work activities, limited outdoor exposure, and a greater prevalence of parental myopia in urban populations. Importantly, China’s current myopia prevention and control system still faces notable challenges (9). For example, a substantial proportion of children with refractive errors remain uncorrected, and the effectiveness of screening programs in grassroots regions remains suboptimal, thereby complicating efforts to manage myopia effectively. Consequently, the establishment of a standardized campus-based screening mechanism is both urgent and critical. Early detection coupled with timely intervention can serve as an effective strategy to mitigate this public health challenge.

To date, China has established province-level public health frameworks for myopia containment across diverse geographical regions, with the health-education partnership paradigm achieving substantial progress in multiple municipalities (10). However, substantial disparities continue to exist in the effectiveness of myopia prevention and control across various geographic regions. Accumulating epidemiological evidence indicates that the myopia incidence rate among children in Beijing reaches 35.1% (11), in contrast to 22.4% reported in rural areas of Hebei Province (12). Xingtai, a medium-sized city located in North China, occupies an intermediary position between major urban centers and rural regions, thereby displaying typical characteristics of an urban–rural transitional zone. Despite growing attention to myopia prevention, research specifically investigating the prevalence and associated risk factors among children in such transitional areas remains insufficient.

Furthermore, spherical equivalent (SE) has historically served as the primary parameter for clinical myopia screening in epidemiological and clinical studies. However, SE merely reflects the end-point refractive status and lacks the capacity to distinguish the individual contributions of corneal curvature (CR) and axial length (AL) to the progression of myopia. In contrast, these biometric parameters—particularly AL and CR—are more sensitive indicators of pathological structural changes in the eye. Therefore, the International Myopia Institute (IMI) (13) recommends integrating biometric parameters such as AL and CR into standardized myopia screening protocols. This approach enables more accurate stratification of myopia-related risks and facilitates targeted preventive interventions, thereby enhancing both the precision and clinical effectiveness of myopia management strategies.

Building upon the aforementioned context, this study employed cross-sectional data from myopia screening conducted among primary school students in the Xingtai region. The research innovatively incorporated key ocular biometric parameters, including corneal curvature and axial length, to systematically investigate the associations between refractive components and visual development. The results provide robust, evidence-based support for the formulation of a regionally tailored framework for precision prevention and control of myopia.

## Patients and methods

### Research subjects

This study utilized a cluster sampling design to determine the required sample size, based on the “National Student Physical Health Standard” (GB/T 26343–2010) and regional epidemiological data. The minimum sample size was calculated using a formula adjusted for design effect (design effect = 1.5), which incorporated a 95% confidence level, a 2% margin of error, and an estimated myopia prevalence of 35%. The calculation indicated that a minimum of 11,600 participants would be required.

The sampling procedure was conducted during the 2022–2023 academic year and followed a multi-stage cluster sampling approach. First, based on the administrative divisions of Xingtai City, the study area was divided into 18 geographical strata. Subsequently, 11 central primary schools, representative of both urban and rural settings, were selected as primary sampling units using the Probability Proportionate to Size (PPS) sampling method. Within each selected school, stratified cluster sampling was implemented to ensure the random selection of at least three natural classes per grade level. A total of 245 standardized classes were ultimately designated as the basic sampling units. Data on all registered students enrolled during the study period and meeting the age criteria of 6–12 years were obtained through access to the education management platform of the local education bureau.

Ultimately, 13,419 individuals aged 6–12 years were enrolled in the survey, surpassing the minimum sample size and thereby enhancing statistical power. To further ensure data integrity, a 10% buffer was included to accommodate potential invalid responses. Incomplete screening in a subset of participants led to the exclusion of 1,394 cases with missing data or outliers (680 males and 714 females). As a result, 12,025 subjects who completed all ophthalmic examinations were included in the statistical analysis.

Ethical approval for this study was obtained from the Institutional Review Board (IRB) of Aier Eye Hospital (approval number: AIER-EC-2024-043), which strictly adheres to the ethical standards articulated in the Declaration of Helsinki. Legally valid informed consent forms were obtained from all participants’ authorized guardians, verifying complete understanding of the research aims, methodological framework, and data management protocols. Throughout the research duration, participants’ core rights—specifically voluntary participation and the right to withdraw at any stage without penalty—were institutionally protected in compliance with international research ethics guidelines.

### Procedure

In accordance with the “Standard Logarithmic Visual Acuity Chart” (GB11533-2011), distant visual acuity was evaluated. For children and adolescents aged 6 years and older, a visual acuity of less than 5.0, as measured by the five-point recording method, is officially categorized as impaired vision under China’s mandatory health standard WS/T 793-2022. Specifically, visual acuity of 4.9 was categorized as mild visual impairment, a range of 4.6–4.8 as moderate visual impairment, and ≤4.5 as severe visual impairment. Under non-cycloplegic conditions, screening myopia was defined as SE < −0.50D, where SE = spherical component (S) + 1/2 cylindrical component (C). Participants were stratified into two groups based on corrected visual acuity: suboptimal correction (<4.9) and adequate correction (≥4.9). The proportions of participants with adequate correction and suboptimal correction were subsequently calculated.

The basic information of the subjects, as well as parameters related to ophthalmology and optometry (standardized visual acuity testing, refractive error assessment, and ocular biometric measurement), were systematically collected. Quality control measures are structured across three core domains: personnel qualifications, equipment calibration, and operational protocols. All operators are required to hold standardized optometry certifications and undergo daily consistency checks, with inter-operator agreement assessed via Kappa statistics (Kappa value > 0.85). Equipment accuracy is validated each day prior to use by a mobile calibration laboratory employing standard spherical lenses (±5D) and cylindrical lenses (±2D@180°), ensuring measurement error remains below 0.25D. Refractive measurements are performed by certified optometrists using the NIDEK AR-1 automatic refractometer under non-cycloplegic conditions. Environmental lighting is strictly controlled to maintain a minimum pupil diameter of 2 mm, and equipment height is adjusted according to the subject’s physical stature. Each eye is measured three times, and any measurement deviating from the other two by more than the allowable threshold is repeated. The final data used for statistical analysis are derived from the average of the three valid measurements. For ocular biometric examination, the German ZEISS IOL Master 700 optical biometer was utilized to measure parameters such as axial length (AL), keratometry values (*K*₁, *K*₂), and calculate the *K_m_* (*K_m_* = (*K*_1_ + *K*_2_)/2) and axial-to-corneal ratio (AL/CR).

### Statistical analysis

Statistical analyses were performed using SPSS 25.0. All measurement data underwent normality testing and homogeneity of variance assessment. Data conforming to a normal distribution were expressed as mean ± standard deviation. Scatter plots and Pearson correlation analysis were utilized to evaluate the consistency of SE, average K, AL, and AL/CR between both eyes. In this study, one-way analysis of variance (one-way ANOVA) and Pearson’s chi-square test were used to evaluate intergroup differences in various parameters across different grade levels. All hypothesis tests were carried out using two-tailed *p*-values, with statistical significance predetermined at *α* = 0.05. A *p*-value less than 0.05 was considered statistically significant. To explore the relationship between AL/CR and SE, both linear and nonlinear regression models were applied. Three fundamental models—the linear regression model, the quadratic polynomial regression model, and the logarithmic regression model—were established. Model performance was evaluated using the adjusted coefficient of determination (adjusted *R*^2^), which supported the selection of the best-fitting model. The mathematical relationship between AL/CR and SE was then derived based on the selected model.

## Results

**Figure 1:**
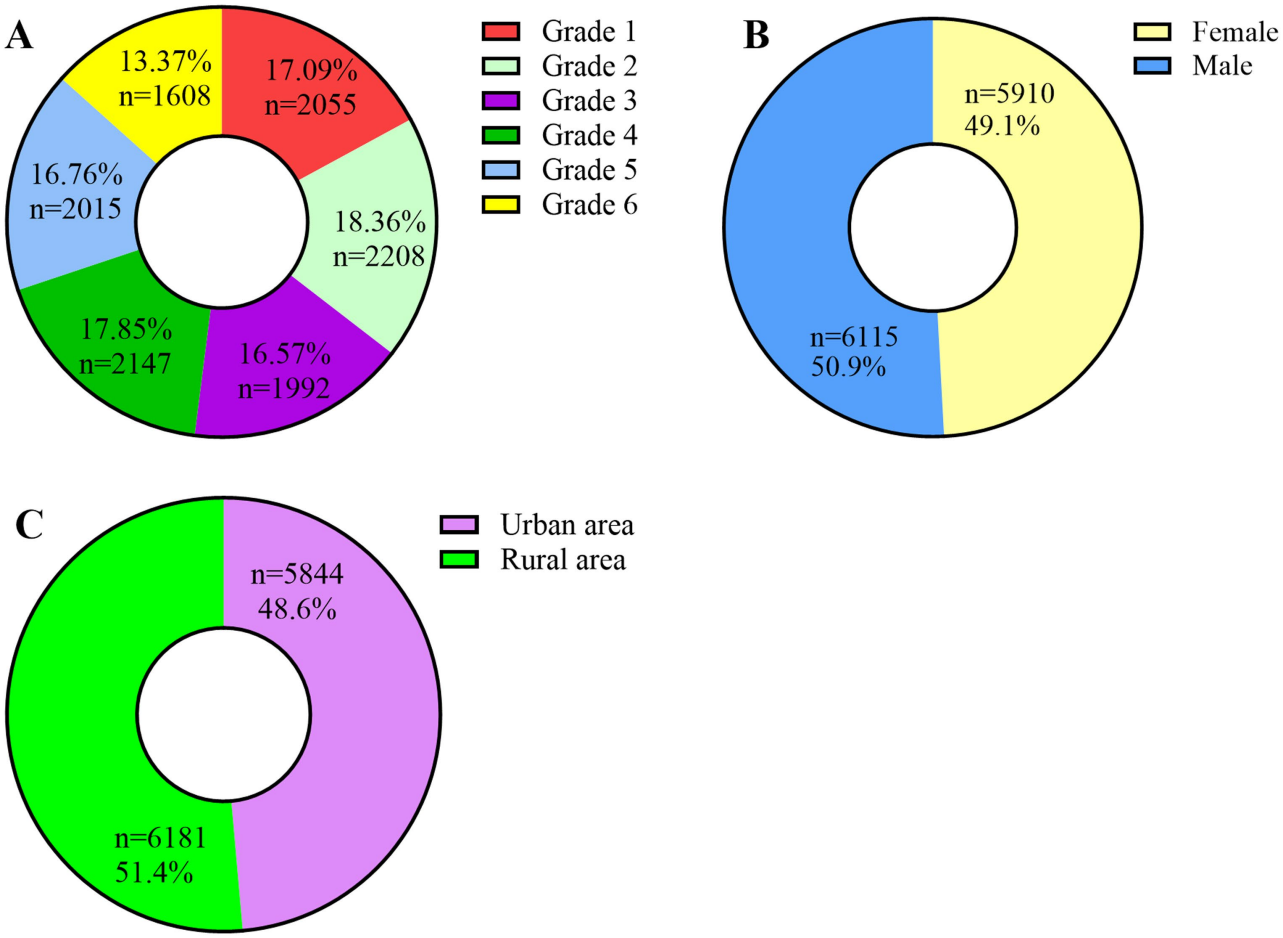
**(A)** Distribution of student population by grade level; **(B)** Distribution of student population by gender; **(C)** Distribution of student population by urban–rural geographic location.

**Figure 2:**
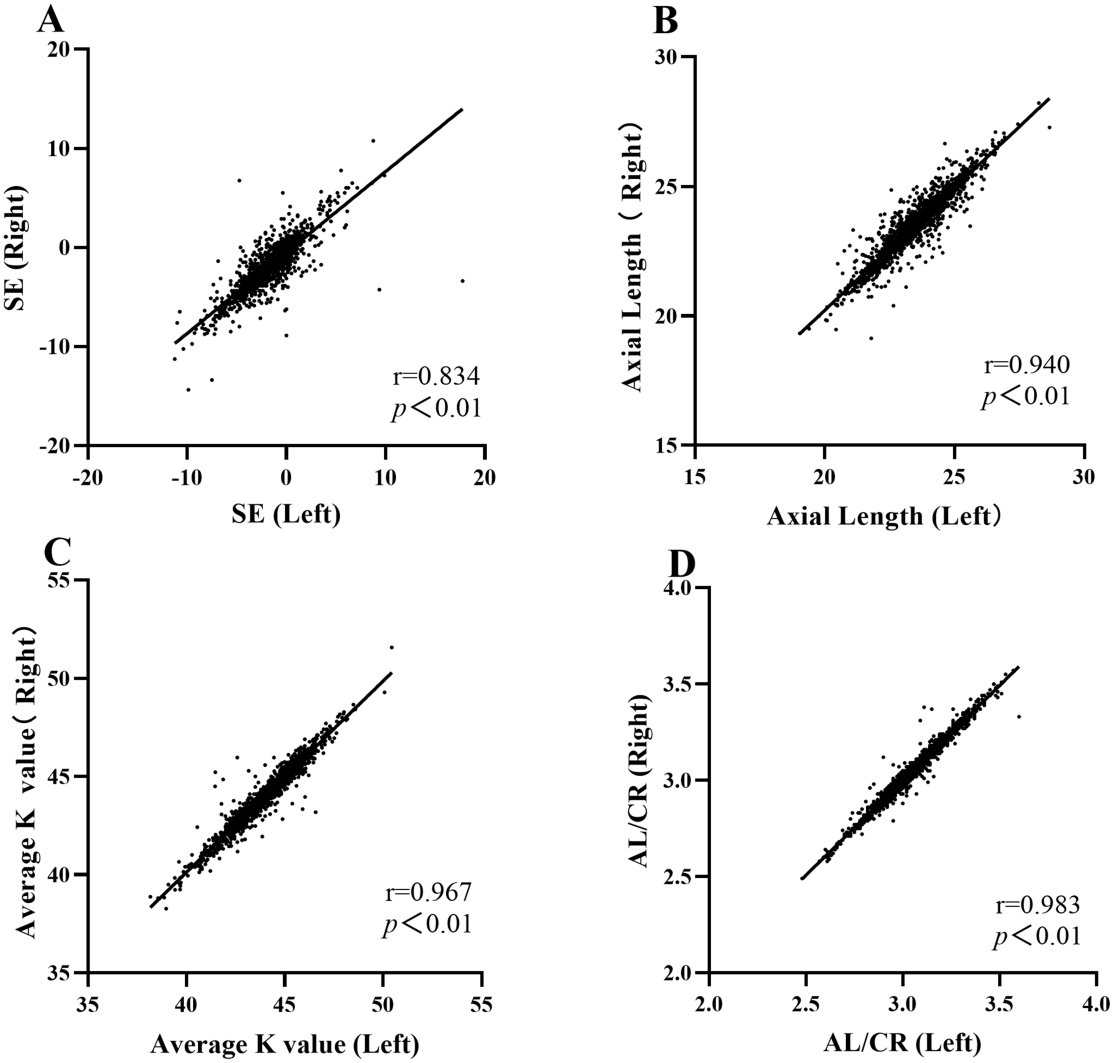
**(A)** Pearson correlation analysis of right vs. left eye datasets on Spherical Equivalent (SE). **(B)** Pearson correlation analysis of right vs. left eye datasets on Axial Length (AL). **(C)** Pearson correlation analysis of right vs. left eye datasets on Average Keratometry Value. **(D)** Pearson correlation analysis of right vs. left eye datasets on AL/CR.

**Figure 3:**
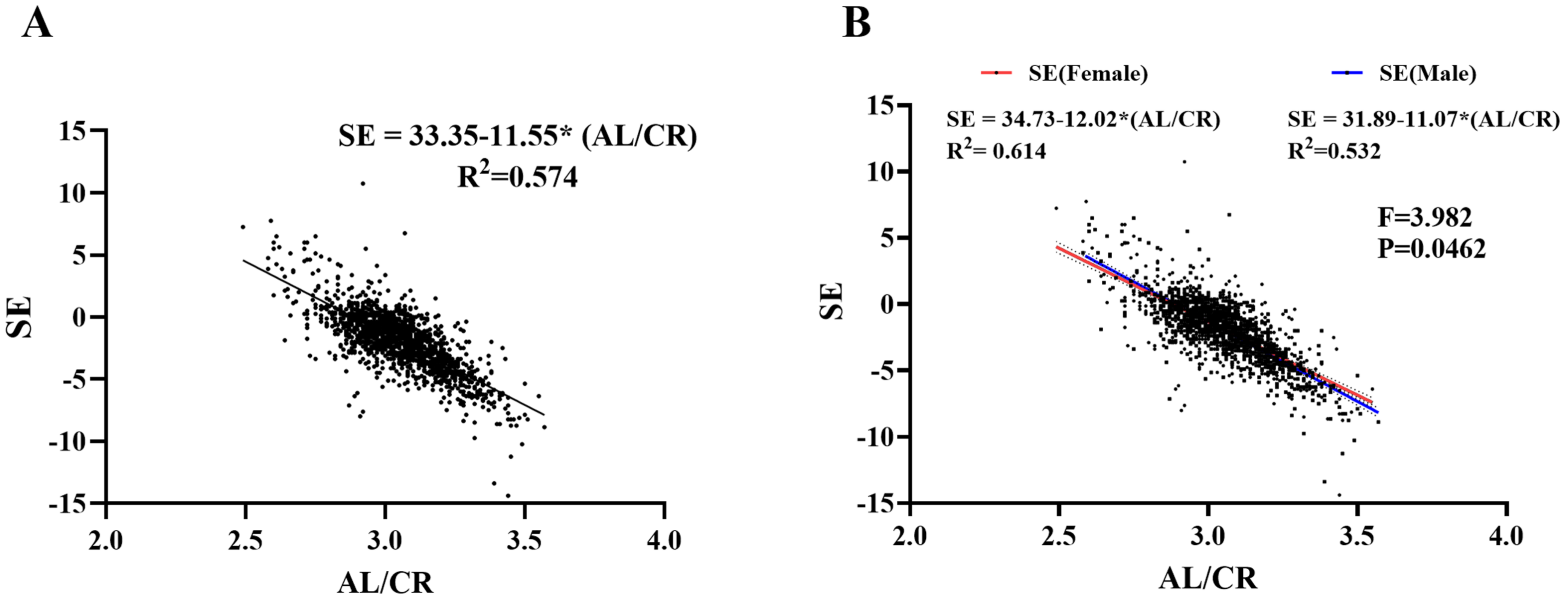
**(A)** Linear regression analysis of SE on AL/CR across the entire study population. **(B)** Gender-specific linear regression analysis of SE on AL/CR in the study cohort.

1. The study cohort consisted of 12,025 primary school students, including 6,115 males (50.9%) and 5,910 females (49.1%), who were recruited from 11 educational institutions in Xingtai City, Hebei Province. Baseline demographic characteristics, with respect to academic progression, were as follows: Grade 1 (*n* = 2,055; 17.1%), Grade 2 (*n* = 2,208; 18.4%), Grade 3 (*n* = 1,992; 16.6%), Grade 4 (*n* = 2,147; 17.9%), Grade 5 (*n* = 2,015; 16.8%), and Grade 6 (*n* = 1,608; 13.4%). With regard to geographic distribution, 5,844 students (48.6%) were from urban areas and 6,181 students (51.4%) were from rural areas (Figure 1).
2. Pearson correlation analyses were independently conducted on the right and left eye datasets. (SE: *r* = 0.834, *p* ≤ 0.001; AL: *r* = 0.940, *p* ≤ 0.001; Average *K* value: *r* = 0.967, *p* ≤ 0.001; AL/CR: *r* = 0.983, *p* ≤ 0.001) (Figure 2). The statistical results revealed a strong correlation and high consistency between the two datasets. Consequently, the right eye dataset was selected for subsequent statistical analysis to serve as a representative sample of the overall findings.
3. Screening results: The results demonstrated that the detection rate of impaired visual acuity was 65.65% (7,895 out of 12,025 participants). A statistically significant increase in prevalence was observed with advancing school grades, with rates rising from 58.00% in Grade 1 to 75.75% in Grade 6. The prevalence of screening-defined myopia reached 51.69%, showing a progressive increase across grades, ranging from 30.41% in Grade 1 to 68.78% in Grade 6. Refractive parameter analysis revealed a progressive myopic shift in SE values, which decreased from −0.66 ± 1.54 D in Grade 1 to −2.20 ± 2.01 D in Grade 6, with an overall mean of −1.46 ± 1.84 D. AL measurements showed a consistent increase across grades, progressing from 22.95 ± 0.83 mm in Grade 1 to 24.13 ± 1.12 mm in Grade 6, with an overall mean of 23.64 ± 1.07 mm. *K*-value remained relatively stable at 43.39 ± 1.51 D, with inter-grade variation not exceeding 0.13 D (ranging from 43.33 D to 43.46 D). Furthermore, AL/CR exhibited a significant trend of progression across grades, increasing from 2.95 ± 0.10 in Grade 1 to 3.08 ± 0.14 in Grade 6, with an overall mean of 3.02 ± 0.17. The detailed results are presented in Table 1.
4. To elucidate the association between AL/CR and SE, this study developed three foundational regression models—linear, quadratic polynomial, and logarithmic—and systematically evaluated their goodness-of-fit. The adjusted coefficients of determination (adjusted *R*^2^) for these models were 0.578, 0.574, and 0.569, respectively, indicating that the linear model exhibited superior explanatory power. Consequently, the linear regression model was selected for further analysis. The derived regression equation for the overall study population was SE = 33.35–11.55 × (AL/CR) (Figure 3A). To investigate potential gender-specific variations in the relationship between AL/CR and SE, stratified analyses by gender were conducted, and separate linear regression models were fitted for males and females. The resulting equations were SE = 34.73–12.02 × (AL/CR) for males and SE = 31.89–11.07 × (AL/CR) for females. A comparative analysis of these gender-specific models revealed statistically significant differences in regression slopes, as evidenced by the *F*-test results (*F* = 3.982, *p* = 0.0462) (Figure 3B).

**Table 1:** Screening results of enrolled students.

Characteristics	Poor vision	Vision correction	Adequately correction	Under correction	Myopia prevalence	Astigmatism	UCVA (D)	SE (D)	Cyl (D)	AL (mm)	K (D)	AL/K
Grade1	1129(58.0%)	122 (10.8%)	36 (29.51%)	86 (70.49%)	625 (30.41%)	525 (25.55%)	4.88 ± 0.18	0.66 ± 1.54	0.51 ± 0.61	22.95 ± 0.83	43.43 ± 1.44	2.95 ± 0.10
Grade2	1199(54.3%)	237 (19.77%)	84 (35.44%)	153 (64.56%)	581 (38.54%)	625 (28.31%)	4.86 ± 0.23	1.01 ± 1.52	0.54 ± 0.62	23.24 ± 0.89	43.46 ± 1.73	2.99 ± 0.10
Grade3	1291(64.8%)	303 (23.47%)	93 (30.69%)	210 (69.31%)	1,037 (52.06%)	611 (30.67%)	4.80 ± 0.27	1.35 ± 1.69	0.57 ± 0.62	23.56 ± 0.98	43.42 ± 1.44	3.02 ± 0.11
Grade4	1492(69.5%)	483 (32.37%)	171 (35.40%)	312 (64.60%)	1,262 (58.78%)	735 (34.23%)	4.73 ± 0.32	1.67 ± 1.90	0.77 ± 0.71	23.83 ± 1.04	43.33 ± 1.46	3.05 ± 0.13
Grade5	1503(74.6%)	586 (38.99%)	197 (33.6%)	389 (66.38%)	1,335 (66.2%)	796 (39.5%)	4.68 ± 0.34	2.04 ± 1.92	0.68 ± 0.75	24.06 ± 1.07	43.34 ± 1.45	3.07 ± 0.13
Grade6	1218(75.8%)	472 (38.75%)	166 (35.17%)	306 (64.83%)	1,106 (68.78%)	655 (40.73%)	4.65 ± 0.35	2.20 ± 2.01	0.69 ± 0.71	24.13 ± 1.12	43.35 ± 1.53	3.08 ± 0.14
Total/mean	7895(65.7%)	2,203 (27.90%)	747 (33.91%)	1,456 (66.09%)	6,216 (68.78%)	3,947 (32.82%)	4.77 ± 0.30	1.46 ± 1.84	0.60 ± 0.65	23.64 ± 1.07	43.39 ± 1.51	3.02 ± 0.17
*X^2^/F*	385.24	391.88	3.540	54.883	112.468	162.304	223.889	222.705	23.413	440.603	2.947	395.941
*p*	≤0.001	≤0.001	0.617	≤0.001	≤0.001	≤0.001	≤0.001	≤0.001	≤0.001	≤0.001	0.012	≤0.001

UCVA, uncorrected visual acuity; SE, spherical equivalent; Cyl, cylindrical diopter; AL, axial length; K, keratometry value.

## Discussion

Refractive error is a leading cause of visual developmental disorders in children (2) and represents one of the most pressing public health challenges currently facing China, with the situation continuing to deteriorate. In recent years, myopia prevention and control strategies in China have increasingly emphasized prevention over correction. This study focuses on the critical period of 6–12 years for children’s myopia development and establishes a standardized, school-based vision screening system. The screening results indicate that the prevalence of poor vision reaches as high as 65.65%, with a pronounced upward trend observed as grade levels increase. Key contributing factors include the escalating academic burden associated with higher grades, prolonged near-work activities, and diminished outdoor activity, all of which exacerbate vision-related issues. Notably, first-grade students exhibit a higher prevalence of poor vision compared to second-grade students (58% vs. 54.03%, *p* < 0.001). This disparity may stem from the incomplete maturation of visual development in younger children. Furthermore, current screening standards are based on adult norms for normal vision, which may result in an underestimation of vision capabilities among some children. Therefore, it is recommended to incorporate a ciliary muscle function monitoring module into the existing screening framework and integrate it with a dynamic refractive profile tracking mechanism to enhance the accuracy of evaluating and managing children’s vision health.

This study identifies intermediate transitional patterns in the prevalence trends of myopia. The observed regional variations in myopia prevalence appear to be closely associated with factors including geographical distribution, urban–rural structure, and levels of economic development (9). The results reveal a screening myopia rate of 51.69% among the entire cohort of children, which is significantly higher than the 32% reported in the UK (14), yet lower than the 63.1% documented in developed cities in eastern China (15). Within the broader North China region, the myopia rate in Xingtai is markedly higher than both the 22.4% observed in rural areas of Hebei Province (12) and the 35.1% reported in Beijing, a megacity in the same region (11). This pronounced regional disparity suggests that Xingtai, as a medium-sized city positioned between major urban centers and rural areas, may confront a “double disadvantage “in the domain of myopia prevention and control. An analysis of the underlying causes demonstrates that rural areas may experience “passive protection” against myopia due to children’s prolonged outdoor activity time. In contrast, large cities, supported by concentrated policy resources, achieve effective myopia prevention and control through proactive interventions, such as the standardization of classroom lighting, the implementation of regular vision screening systems, and increased parental awareness regarding visual health. Compared with these two models, medium-sized cities often remain inadequately addressed by either strategy. The findings of this study further illustrate that the elevated myopia incidence in cities like Xingtai accurately reflects their transitional phase within the urbanization process, which is marked by underdeveloped health intervention mechanisms. This underscores the practical significance and innovative value of the present research in advancing regional myopia prevention and control strategies.

Longitudinal data analysis indicates a pronounced grade-dependent increase in myopia prevalence, rising from 30.41% in the first grade to 68.78% in the sixth grade. Notably, the most substantial increase in myopia prevalence occurs in the third grade, suggesting that this period may serve as a critical window for myopia prevention and control among students. Therefore, early intervention appears to be particularly crucial (16). Based on these findings, it is recommended that relevant authorities and parents prioritize this period by designing and implementing effective measures to further enhance vision protection efforts.

Therefore, grounded in the aforementioned research findings and prevailing societal conditions, this study proposes the development of a systematic and multi-tiered intervention framework for the prevention and control of childhood myopia. The specific recommendations are as follows: Medium-sized cities should be prioritized as focal intervention zones, coordinated by provincial health administrative authorities. Dedicated financial resources should be allocated to enhance critical infrastructure that can yield measurable improvements in the short term, including the standardization of classroom lighting and the ergonomic adjustment of desks and chairs. Regional educational and health authorities are encouraged to design and implement context-specific “fragmented outdoor activity” programs aligned with local academic calendars. This strategic approach is intended to improve both the operational feasibility of policy implementation and the behavioral compliance of students. Concurrently, it is imperative to strengthen the science communication infrastructure for myopia prevention and control. This would not only enhance public awareness but also foster greater community engagement and proactive participation in comprehensive childhood myopia management initiatives.

A survey of the refractive correction status among 7,895 students with suboptimal vision revealed that the spectacle correction rate was 27.9%. Among those who had been corrected, the adequately correction rate was 33.91%, whereas the under-correction rate was as high as 66.09%. This disparity may be attributed to limited awareness of myopia and the significance of refractive correction among parents and students, including delayed recognition of vision changes, reluctance to undergo professional refraction, and misconceptions about spectacle correction (e.g., the belief that wearing glasses accelerates myopia progression). Moreover, both uncorrected and under-corrected conditions can accelerate axial elongation through the retinal defocus mechanism, potentially leading to further vision deterioration. In contrast, standardized correction has been demonstrated to significantly reduce the progression rate of myopia (17). For groups with low acceptance of spectacle correction, orthokeratology lenses are recommended as an alternative intervention. Orthokeratology lenses enhance daytime uncorrected visual acuity via nighttime wear, thereby improving the quality of life for adolescents while effectively slowing myopia progression (18).

This study revealed that the prevalence of astigmatism among the enrolled students was 32.82% (Table 1), lower than the 61.7% reported in northeast Sichuan (19). This disparity may be ascribed to several factors, including the diagnostic criteria for astigmatism adopted in this study (≥0.50D as the inclusion threshold), variations in ocular biological parameters across ethnic groups, and differences in measurement equipment and methodologies (e.g., automatic versus manual refractometers). Furthermore, astigmatism demonstrates significant age-related characteristics, aligning with the general pattern of corneal morphological development during adolescence. However, some studies (20, 21) have drawn opposing conclusions. Such discrepancies may arise from differences in the refractive status of participants (e.g., changes in astigmatism associated with myopia progression), measurement protocols (e.g., whether cycloplegia was performed), and regional environmental factors. Consequently, it is clear that the evolution of astigmatism is influenced by multiple factors. Future research should focus on establishing a unified diagnostic standard and conducting multi-center longitudinal cohort studies to further investigate its biological mechanisms and environmental regulatory factors. Additionally, incorporating advanced detection methods, such as corneal topography, could enhance the scientific rigor and reliability of the research.

Axial myopia represents a dominant form of myopia in children and adolescents. As axial length increases and exposure duration extends, the risk of fundus complications markedly rises (22). Therefore, slowing the rate of axial elongation is essential for reducing the risk of fundus complications associated with high myopia. In this study, the overall mean axial length was 23.64 ± 1.07 mm, showing a clear gradient increase across grades (Grade 1: 22.95 ± 0.83 mm vs. Grade 6: 24.13 ± 1.12 mm), with statistically significant differences (ANOVA: *F* = 440.603, *p* < 0.001). These results are highly consistent with data from a large-scale cohort study in Shandong Province (6 years: 22.65 ± 0.76 mm; 12 years: 24.14 ± 1.01 mm) (23).

The AL/CR offers a more accurate reflection of the degree of emmetropization compared to AL or CR, and exhibits enhanced predictive value in studies of childhood myopia (24–26). This study observed a statistically significant increase in AL/CR across grade levels (*F* = 395.941, *p* < 0.001), a trend consistent with findings reported by Yebra et al. (25) and Mu et al. (26) Linear regression analysis revealed a significant association between AL/CR and SE, with the regression equation: SE = 33.35–11.55 × (AL/CR). These results indicate that as AL/CR increases, SE decreases linearly, underscoring AL/CR as a key predictive factor for the onset and progression of childhood myopia. Consequently, AL/CR may serve as a reliable biomarker for monitoring myopia development and provide a solid quantitative foundation for establishing a myopia risk prediction model.

Subsequently, the screened children were stratified into male and female subgroups for detailed subgroup analysis. The results demonstrated that both groups exhibited significant linear regression relationships; however, a statistically significant difference was observed in the regression slopes (*p* < 0.05). Specifically, the male subgroup exhibited a steeper slope, suggesting greater sensitivity to variations in the AL/CR ratio. An increase in AL/CR was typically accompanied by a rapid decline in SE values, indicating that AL/CR may have a higher predictive value for refractive state changes in males compared to females. To explore the underlying mechanisms, it is hypothesized that these differences may be associated with variations in axial length growth rates during development between the two genders. These gender-specific physiological characteristics could be closely related to sex hormone-mediated regulation of scleral remodeling, differential expression of the insulin-like growth factor signaling pathway across genders, and gender-specific sensitivity of the retinal defocus compensation mechanism. Such distinct patterns in axial length development may reflect a differentiated physiological response mechanism that has evolved within the human visual system in response to gender-related environmental adaptation pressures during phylogenetic development (27–30).

A methodological limitation stems from the utilization of non-cycloplegic computerized refraction in mass screening protocols, which could induce refractive measurement inaccuracies and inflate prevalence estimates of screen-detected myopia. As this was a cross-sectional study, the observed associations require further validation through longitudinal cohort investigations. The cross-sectional design inherently limits the ability to determine whether the observed differences across age groups reflect genuine incidence accumulation or are influenced by cohort effects arising from historical shifts in educational practices. Nevertheless, this cross-sectional investigation has successfully established a baseline refractive development database for school-aged populations in Xingtai. Longitudinal tracking based on this platform provides an epidemiological framework for elucidating region-specific patterns of myopia progression. This infrastructure will further enable the formulation of multidimensional intervention models incorporating genetic predisposition analysis and environmental exposure profiling.

Childhood myopia constitutes not only a clinical condition but a significant public health challenge bearing substantial socioeconomic implications for national development. The pathophysiological pathways underlying myopia progression remain incompletely elucidated, with persistent controversies surrounding its multifactorial origins. While hereditary predisposition has been conclusively identified as a principal determinant, environmental modulators exert critical influence in mediating both the initiation and advancement of refractive error in genetically susceptible populations. The implementation of school-based refractive screening programs, coupled with the development of population-level refractive surveillance systems and longitudinal risk factor tracking, emerges as priority public health interventions for early detection and targeted management of high-risk cohorts. Parallel initiatives should encompass comprehensive health education campaigns to elevate preventive awareness among stakeholders and the adoption of clinically validated ocular hygiene protocols, collectively forming an evidence-driven framework for mitigating myopia prevalence and preserving adolescent visual wellbeing.

## Data Availability

The data analyzed in this study is subject to the following licenses/restrictions: The datasets are not publicly available due to privacy/ethical restrictions (e.g., participant confidentiality under China’s Personal Information Protection Law), but anonymized data may be available from the corresponding author upon reasonable request and with permission from the Xingtai Municipal Education Bureau. Requests to access these datasets should be directed to YL, hbykdxdyyyyklyy@hebmu.edu.cn.
